# Transdermal Uptake of Diethyl Phthalate and Di(*n*-butyl) Phthalate Directly from Air: Experimental Verification

**DOI:** 10.1289/ehp.1409151

**Published:** 2015-04-07

**Authors:** Charles J. Weschler, Gabriel Bekö, Holger M. Koch, Tunga Salthammer, Tobias Schripp, Jørn Toftum, Geo Clausen

**Affiliations:** 1 Environmental and Occupational Health Sciences Institute, Rutgers University, Piscataway, New Jersey, USA; 2 International Centre for Indoor Environment and Energy, Department of Civil Engineering, Technical University of Denmark, Lyngby, Denmark; 3 Institute for Prevention and Occupational Medicine of the German Social Accident Insurance, Institute of the Ruhr-Universität Bochum (IPA), Bochum, Germany; 4 Department of Material Analysis and Indoor Chemistry, Fraunhofer WKI, Braunschweig, Germany

## Abstract

**Background:**

Fundamental considerations indicate that, for certain phthalate esters, dermal absorption from air is an uptake pathway that is comparable to or greater than inhalation. Yet this pathway has not been experimentally evaluated and has been largely overlooked when assessing uptake of phthalate esters.

**Objectives:**

This study investigated transdermal uptake, directly from air, of diethyl phthalate (DEP) and di(*n*-butyl) phthalate (DnBP) in humans.

**Methods:**

In a series of experiments, six human participants were exposed for 6 hr in a chamber containing deliberately elevated air concentrations of DEP and DnBP. The participants either wore a hood and breathed air with phthalate concentrations substantially below those in the chamber or did not wear a hood and breathed chamber air. All urinations were collected from initiation of exposure until 54 hr later. Metabolites of DEP and DnBP were measured in these samples and extrapolated to parent phthalate intakes, corrected for background and hood air exposures.

**Results:**

For DEP, the median dermal uptake directly from air was 4.0 μg/(μg/m^3^ in air) compared with an inhalation intake of 3.8 μg/(μg/m^3^ in air). For DnBP, the median dermal uptake from air was 3.1 μg/(μg/m^3^ in air) compared with an inhalation intake of 3.9 μg/(μg/m^3^ in air).

**Conclusions:**

This study shows that dermal uptake directly from air can be a meaningful exposure pathway for DEP and DnBP. For other semivolatile organic compounds (SVOCs) whose molecular weight and lipid/air partition coefficient are in the appropriate range, direct absorption from air is also anticipated to be significant.

**Citation:**

Weschler CJ, Bekö G, Koch HM, Salthammer T, Schripp T, Toftum J, Clausen G. 2015. Transdermal uptake of diethyl phthalate and di(*n*-butyl) phthalate directly from air: experimental verification. Environ Health Perspect 123:928–934; http://dx.doi.org/10.1289/ehp.1409151

## Introduction

Selected phthalates have been associated with multiple health effects, including children’s neurodevelopment (Kim Y et al. 2011; Whyatt et al. 2012), altered genital development (Swan et al. 2005), respiratory problems (Jaakkola and Knight 2008), oxidative stress (Ferguson et al. 2011), obesity (Hatch et al. 2010), and the development of diabetes (Svensson et al. 2011). Phthalates are used in a wide variety of consumer products (Dodson et al. 2012), and their metabolites are commonly found in human urine (Koch et al. 2011; Langer et al. 2014; Zota et al. 2014). Several studies have assessed the relative contribution of diet, dust ingestion, inhalation, or dermal absorption to the total level of selected phthalates in humans (Bekö et al. 2013; Gaspar et al. 2014; Guo and Kannan 2011; Koch et al. 2013; Wittassek et al. 2011; Wormuth et al. 2006). Prior to 2012, assessments of the dermal pathway focused on contact transfer, including transfer from dust deposited on skin. Kissel (2011) argued that dermal absorption has been underestimated in many studies that have used an inappropriate metric (percent absorption) to assess dermal uptake. Prompted by his critique, there has been recent interest in dermal absorption of phthalates and other semivolatile organic compounds (SVOCs) directly from air (Weschler and Nazaroff 2012, 2014; Gong et al. 2014b). Numerous experimental studies have demonstrated that certain volatile organic compounds (VOCs) are dermally absorbed from air at rates that are comparable to their intake via inhalation (e.g., Bader et al. 2008; Piotrowski 1967, 1971; other references in Weschler and Nazaroff 2014). Although fundamental physical–chemical considerations indicate that the same should be true for certain SVOCs, we are aware of no investigations that have measured direct dermal uptake from air for any SVOC.

Diethyl phthalate (DEP) and di(*n*-butyl) phthalate (DnBP) are among the SVOCs predicted to have substantial dermal uptake directly from air (Weschler and Nazaroff 2012, 2014). DEP and DnBP belong to the group of so-called low-molecular-weight phthalates with two and four carbon atoms in the alkyl chain, respectively. Their chemical and physical properties are summarized in Supplemental Material, Table S1. DEP is used in personal care products such as cosmetics, perfumes, and shampoos; it is also used in the automotive sector and food packaging. DnBP is a common ingredient of adhesives and coatings and is used as a solvent for organic compounds, as an antifoam agent, as a fiber lubricant, and as an additive in cosmetics, including nail polish (Bolgar et al. 2008; Dodson et al. 2012; Wypych 2012). DEP and DnBP have been identified in indoor air and dust samples from around the world (e.g., Fromme et al. 2004; Rudel et al. 2003; Wensing et al. 2005; Weschler and Nazaroff 2008). Their metabolites are often the most abundant synthetic chemicals identified in human urine (Koch et al. 2011; Langer et al. 2014; Zota et al. 2014). The aim of the present study was to experimentally evaluate transdermal uptake of DEP and DnBP directly from the gas phase based on their metabolites in urine. This was accomplished by differential inclusion and exclusion of the inhalation pathway during repeated controlled exposures of human participants in a large chamber.

## Methods

*Human participants*. Six healthy males, 27–66 years of age, agreed to participate in these experiments. Their physiological parameters are summarized in Supplemental Material, Table S2. None had an identified skin disease or skin that was compromised by a wound. The research protocol was approved by the Capital Region of Denmark Committee for Research Ethics (case no. H-3-2013-196). Participants provided informed consent before participation.

*Exposure experiments*. The six participants were divided into groups 1 and 2, each with three subjects. The exposures occurred from 1000 to 1600 hours on Tuesdays and Wednesdays of successive weeks and followed a crossover design. Group 1 was exposed on Tuesdays, with hoods the first week and without hoods the second week. Group 2 was exposed on Wednesdays, without hoods the first week and with hoods the second week. The participants wore only shorts during their 6-hr exposures. They began a restricted diet and restricted use of personal care products 12 hr before entering the chamber and continued these restrictions until the end of urine collection 66 hr later.

*Restricted diet and restricted use of personal care products*. To better distinguish differences in the concentrations of DEP and DnBP metabolites in urine resulting from the chamber exposure, the participants followed a restricted diet and avoided all personal care products from 12 hr before until 54 hr after exposure began. While on the restricted diet, participants were permitted only tap water, tea brewed with tap water, Swedish dried bread, and fruits with thick skins (e.g., bananas, oranges, melons). Avoidance of personal care products included no use of soaps, shampoos, deodorants, colognes, or toothpaste. Preliminary experiments were conducted with eight participants over a 48-hr period to evaluate the efficacy of these restrictions. Background urine concentrations were reduced to medians/means and ranges of 4.1/11.8 and 0.9–53 μg/L for mono-ethyl phthalate (MEP) and 7.4/10.7 and 1.5–37 μg/L for mono-*n*-butyl phthalate (MnBP). The background levels achieved with restrictions were below median background levels in the general population [30 μg/L for MEP and 9.2 μg/L for MnBP for adult males sampled in 2011–2012 (CDC 2015)] and more than two orders of magnitude below peak levels observed in the subsequent exposure experiments.

*Breathing hoods and breathing air*. Supplemental Material, Figure S1, shows one of the authors wearing a breathing hood. The breathing hoods and associated latex neck seals and hoses were purchased from Amron International, Vista, CA (#8890 Oxygen Treatment Hood). Samples cut from each were ultrasonically extracted with acetone and analyzed by GC/MS for both DEP and DnBP using a DB-5MS column and SIM (*m/z* 149); their levels were found to be negligible. The level of another phthalate plasticizer, di(2-ethylhexyl) phthalate (DEHP), was quite high. However, the metabolites of DEHP differ from those of DEP and DnBP (Koch et al. 2013), and do not interfere with the analyses of MEP, MnBP, and 3OH-mono-*n*-butyl phthalate (3OH-MnBP). The breathing air was compressed air that passed through both particulate and activated carbon filters before flowing through the hood at 50 L/min. It was not humidified. Between the first and second round of experiments, duplicate 1-hr air samples were collected from one of the breathing hoods under conditions similar to when the participants were wearing hoods. The measured levels of DEP in the hood with breathing air flowing through were 42.5 and 39.0 μg/m^3^, and those of DnBP were 6.3 and 5.0 μg/m^3^. Because negligible amounts of these phthalates had been measured in the hoods, neck seals, and hoses, we assume that the source of DEP and DnBP was upstream of the hoods. Based on these measurements, all doses reported during the period when the participants were wearing hoods were corrected for inhalation of DEP and DnBP in hood air assuming a breathing rate of 0.7 m^3^/hr (U.S. EPA 2011). Further details are provided below (“Background corrections, hood air corrections, and normalizations”).

*Preparing the chamber for the exposure experiments*. The exposure experiments were conducted at the Technical University of Denmark in a sparsely furnished 55-m^3^ chamber in which air was mixed by two fans. Only one group at a time was exposed in the chamber. Each participant sat on a wire mesh chair at a small table and typically worked on a laptop computer while in the chamber. Supplemental Material, Figure S2, shows group 1 participants in the chamber wearing breathing hoods. The air exchange rate was maintained at 0.7/hr, and the temperature was controlled at 30°C. The relative humidity was not controlled and ranged between 20 and 35% when the participants were in the chamber. Chamber conditions for the experiments are summarized in Supplemental Material, Table S3.

Latex paint, formulated with 1% DEP and 10% DnBP (by weight), was used to deliver these phthalates into chamber air at a relatively constant emission rate. Further details regarding the paint, including experiments conducted to determine the mass fraction of DEP and DnBP required to achieve the targeted steady-state air concentrations, were described by Schripp et al. (2014).

On Monday of the first exposure week, the latex paint spiked with DEP and DnBP was applied on each side of six aluminum plates (1 m × 1 m; total area of 12 m^2^) with a paint roller, and the plates were immediately placed in the chamber. The following Monday the panels were repainted following the same procedure. Each week, during the 6-hr exposures that occurred on Tuesday and Wednesday, the gas-phase concentrations of the two phthalates were determined on an hourly basis. The average gas-phase concentrations measured during the four exposure periods are shown in Table 1, and hourly values measured during the exposure periods are plotted in Supplemental Material, Figure S3. For the exposure periods when hoods were worn, the air concentrations of DEP and DnBP for group 1 were 12% lower than those for group 2, whereas for the exposure periods without hoods, the air concentrations for group 2 were about 20% lower than those for group 1 (Table 1).

**Table 1:** Average concentrations (minimum–maximum) of DEP and DnBP measured in the chamber during the 6-hr exposure intervals.

Day of exposure	Group	Status	DEP (μg/m^3^)	DnBP (μg/m^3^)
Tuesday, 1st week	Group 1	Hoods	250 (240–280)	123 (120–132)
Wednesday, 1st week	Group 2	No hoods	233 (225–252)	114 (108–122)
Tuesday, 2nd week	Group 1	No hoods	291 (282–317)	140 (135–149)
Wednesday, 2nd week	Group 2	Hoods	284 (240–353)	140 (122–163)

*Measurement of phthalate metabolites in urine*. On the mornings of exposure, each participant collected one or two urine samples prior to entering the chamber; after entering the chamber, participants collected all urinations for the next 54 hr—recording the time of the void for each individual sample. Urine voids were collected in 250-mL polypropylene specimen containers known to be phthalate free; during some urinations, more than one vessel was used to collect the urine. Field blanks were not collected, given the many different microenvironments occupied by the participants after leaving the chamber. However, the target analytes—MEP, MnBP, and 3OH-MnBP—are not common contaminants in indoor settings. The samples were weighed the day of collection and then stored in a freezer until they were shipped overnight to the Institute for Prevention and Occupational Medicine of the German Social Accident Insurance (the IPA) in Bochum, Germany. Samples were still frozen upon arrival.

Urine samples were analyzed for MEP, a metabolite of DEP, as well as MnBP and 3OH-MnBP, metabolites of DnBP. The concentrations of these metabolites were determined by two-dimensional high-performance liquid chromatography coupled to tandem mass spectrometry (LC/LC–MS/MS) using internal isotope-labeled standards after enzymatic deconjugation of the phthalate metabolites from the glucuronidated form, following methods published by Koch et al. (2003, 2007) and Preuss et al. (2005). The limits of quantification (LOQs) for MEP, MnBP, and 3OH-MnBP were 0.25, 1.0, and 0.25 μg/L, respectively. Quality control materials, prepared using pooled native urine from various individuals from the IPA to represent the general conjugation status (e.g., glucuronidation) of all urine samples, and reagent blank samples were included in each batch together with the study samples. The laboratory that performed the analysis has successfully participated as a reference lab for phthalate metabolite analyses in the quality assessment/quality assurance program of the European Union (EU)-financed Consortium to Perform Human Biomonitoring on a European Scale (Schindler et al. 2014). The creatinine concentration in urine was measured according to the Jaffé method (Taussky 1954).

*Chemical analysis*. Air samples were collected using stainless-steel tubes filled with Tenax TA (Markes Ltd.) at 100 mL/min (6 L total sampling volume). The tubes were analyzed via thermal desorption (TD100, Markes Ltd.)/gas chromatography (6890 Series GC System, Agilent)/mass spectrometry [5973N MSD (mass selective detector); Agilent] according to DIN ISO 16000-6, (International Organization for Standardization 2012). The GC was equipped with an HP5MS column (60 m × 250 μm × 0.3 μm); the oven temperature program was 30°C (1 min), then increased by 8°C/min to 280°C (5 min). The MSD was operated in selected ion monitoring mode (SIM) using *m/z* 149 (phthalates) and *m/z* 234 (internal standard). Substances were quantified on the basis of original standards. 3-Bromo-biphenyl was used as an internal standard during sampling and analysis. The detection limit for DEP and DnBP as determined from the calibration curve (Einax et al. 1997) was 1 μg/m^3^.

*Calculating uptakes from metabolite concentrations measured in urine*. We calculated the total uptake from the metabolite levels in urine using established procedures (Koch et al. 2007, 2012, 2013). The molecular weights (g/mol) used to convert the metabolites to the parent compounds were as follows: DEP, 222.24; MEP, 194.18; DnBP, 278.34; MnBP, 222.24; and 3OH-MnBP, 238.24. Metabolic conversion factors from metabolism studies (Koch et al. 2012) indicate the fraction of an (oral) dose of parent phthalate that is excreted as a specific metabolite in urine: DEP/MEP, approximately 0.84 (set by analogy to DnBP); DnBP/MnBP, 0.84; and DnBP/3OH-MnBP, 0.07. DnBP uptake was calculated by summing both metabolites:

DnBP (μg) = {[(MnBP/222.24) + (3OH-MnBP/238.24)] × 278.34} ÷ (0.84 + 0.07). [1]

DEP uptake was calculated as

DEP (μg) = [(MEP /194.18) × 222.24] ÷ 0.84. [2]

*Background corrections, hood air corrections, and normalizations*. For each metabolite, we summed the total amount excreted in urine samples collected from the time the participants entered the chamber until the last urine sample was collected about 54 hr later, making no adjustments. Using these values, the total doses of DnBP and DEP were calculated from Equations 1 and 2. The calculated total doses represent both the exposures that occurred during the 6 hr in the chamber as well as those that occurred outside the chamber from 6 to approximately 54 hr. Although participants were on a restricted diet, which limited ingestion of DEP and DnBP, they still inhaled and dermally absorbed “background” DEP and DnBP from the time they left the chamber at 6 hr until their final urine samples were collected at approximately 54 hr. For each participant, we can roughly estimate the dose that occurred in this 48-hr period outside the chamber by using the urine concentrations of metabolites measured in their urine just before entering the chamber coupled with the average volume of urine excreted by each participant in the 48-hr period after leaving the chamber. This gives us an estimate of DEP and DnBP uptake over the 48 hr after leaving the chamber. We subtracted this estimate of “background dose” from the calculated total dose to obtain an estimate of dose during the 6 hr in the chamber. Finally, for the exposures when the participants were wearing hoods, we subtracted the amount of DEP and DnBP inhaled from hood air (170 and 24 μg, respectively). The resulting values represent the dose during exposure in the chamber, corrected for background dose during the period from 6 to 54 hr and also corrected for hood air inhalation intake for the chamber exposures that occurred while participants were wearing hoods. For each participant, the values of the parameters used in these calculations, as well as the final results, are listed in Supplemental Material, Table S4. Finally, for a given 6-hr exposure, total uptakes of DEP and DnBP were normalized first by the chamber air concentrations of DEP and DnBP and then by the participant’s body mass. Although the potential is small, there may be uptake misclassification due to imperfect corrections for “background dose,” hood air, and differences in chamber air concentrations.

## Results

**Figure 1:**
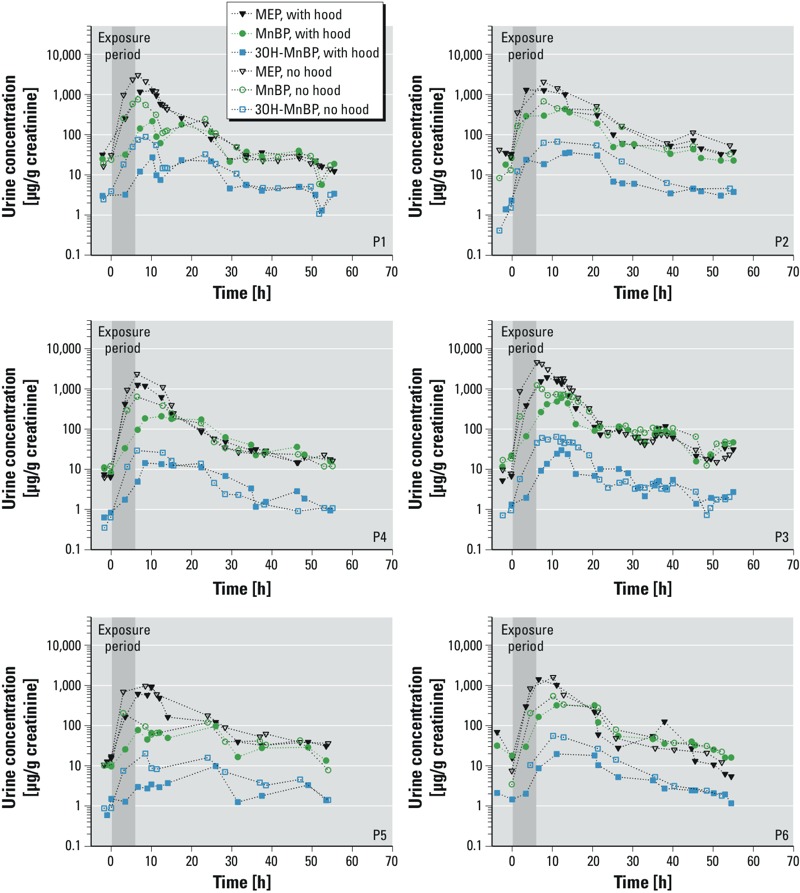
Concentrations (creatinine-normalized) of MEP, MnBP, and 3OH-MnBP in urine samples from the six participants (P1–P6) collected shortly before entering the chamber until 54 hr after the 6-hr exposure began. No adjustments were made to the values other than normalization by creatinine.

*Metabolite concentrations over time*. Figure 1 shows creatinine-normalized concentrations of MEP, MnBP, and 3OH-MnBP in urine samples of each participant collected from the time the participant entered the chamber until 54 hr later (no corrections have been made to the values). When the participants were exposed to elevated chamber concentrations of DEP and DnBP, either wearing a hood or not wearing a hood, urine concentrations of MEP, MnBP, and 3OH-MnBP quickly rose, beginning with the initial urination that occurred after entering the chamber. The metabolite concentrations typically peaked in the first or second urination after the participants exited the chamber after 6 hr exposure, and levels remained elevated with respect to prechamber levels for at least the next 40 hr. Metabolite concentrations were lower when the participants were exposed to chamber air while wearing a hood, but the levels were still substantially higher than levels measured before the participants entered the chamber, indicating significant uptake of DEP and DnBP while participants were wearing a hood.

**Figure 2:**
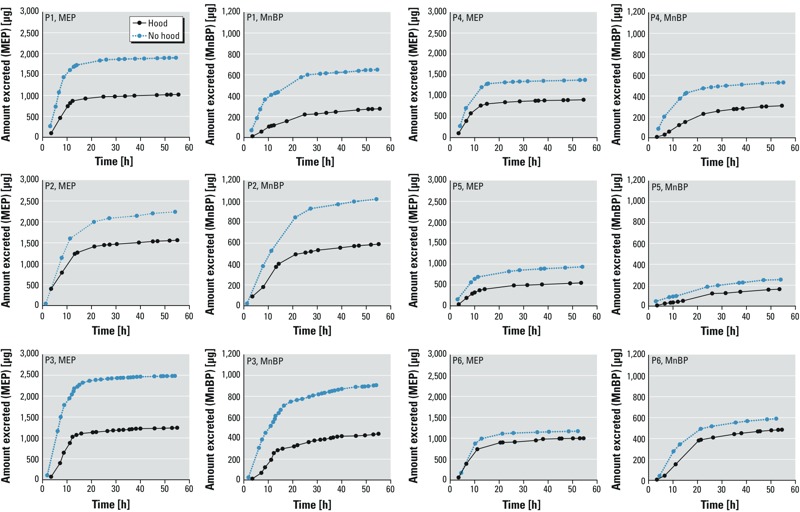
Net amount of MEP and MnBP excreted by each of the six participants (P1–P6) from the time exposure began until 54 hr later. No adjustments were made to the values.

*Net amount of metabolite excreted over time*. For each participant, Figure 2 shows the net amount of MEP and MnBP excreted from the time exposure began until 54 hr later (no corrections have been made to the values). As anticipated, there is participant-to-participant variability. However, for all participants there is substantial excretion of MEP and MnBP when they are exposed while wearing hoods (i.e., when exposure from chamber air is primarily via the dermal pathway). The net amount of MEP excreted when the participants were wearing hoods is roughly half that excreted when not wearing hoods; for MnBP, the fraction is somewhat less than half. It is also apparent from these plots that, during the initial 12 hr, the excreted amounts of both MEP and MnBP increased faster when participants were not wearing hoods than when wearing hoods.

**Figure 3:**
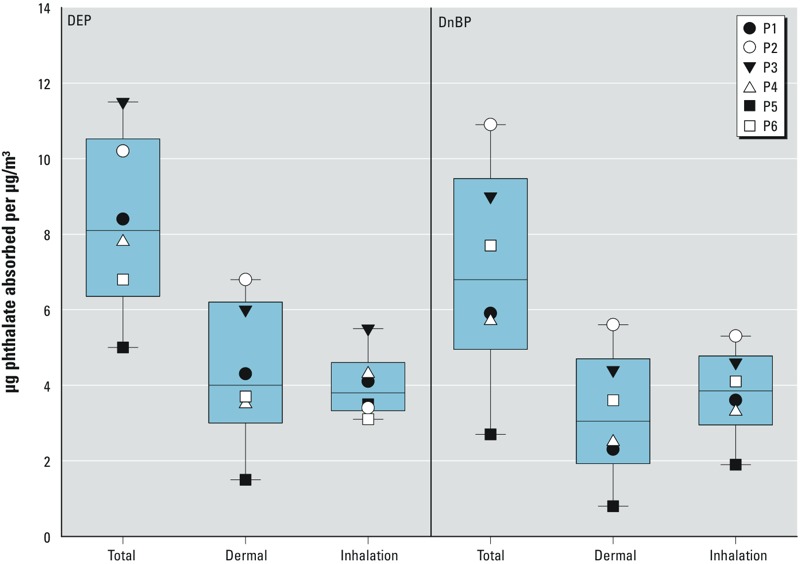
Box plots displaying the uptakes (μg) of DEP and DnBP, corrected for uptakes occurring outside the chamber and from hood air, normalized by measured chamber air concentrations during each exposure experiment (μg/m^3^; Table 1), for exposures without a hood (total) and exposures with a hood (dermal), and for the differences between these two uptakes (inhalation). Boxes extend from the 25th to the 75th percentile, horizontal lines represent the median, and whiskers indicate the 10th and 90th percentiles. The symbols indicate individual results for each of the six participants (P1–P6).

*Total uptake of DEP and DnBP estimated from urinary metabolite levels*. As described in “Methods,” the total uptake of DEP and DnBP can be back-calculated from the concentrations of their metabolites in a participant’s urine samples. Figure 3 displays box plots of the net uptakes (micrograms) of DEP and DnBP, corrected for background and hood air uptakes, and normalized by their measured air concentrations during each exposure experiment (micrograms per cubic meter, Table 1). Separate box plots are displayed for exposures without a hood (total), exposures with a hood (dermal), and the differences between these two uptakes (inhalation). The median value for dermal uptake of DEP is slightly higher than that for its inhalation intake [4.0 vs. 3.8 μg/(μg/m^3^ air)]. The median value for dermal uptake of DnBP is about 80% of that for its inhalation intake [3.1 vs. 3.9 μg/(μg/m^3^ air)].

*Total uptakes normalized by air concentrations and body weight*. The final normalization that we made was to divide the net uptakes in Figure 3 by the body weight of each participant. Supplemental Material, Figure S4, shows a box plot displaying the micrograms of uptake per kilogram of body weight per microgram per cubic meter of air for exposures without a hood (total) and with a hood (dermal) and for the difference (inhalation). When the uptakes are normalized in this manner, the median dermal uptake of DEP is about 10% higher than its inhalation intake (0.048 vs. 0.043 μg uptake/kg body weight per μg/m^3^ air), and the dermal uptake of DnBP is 82% of its inhalation intake (0.040 vs. 0.049 μg uptake/kg body weight per μg/m^3^ air).

**Figure 4:**
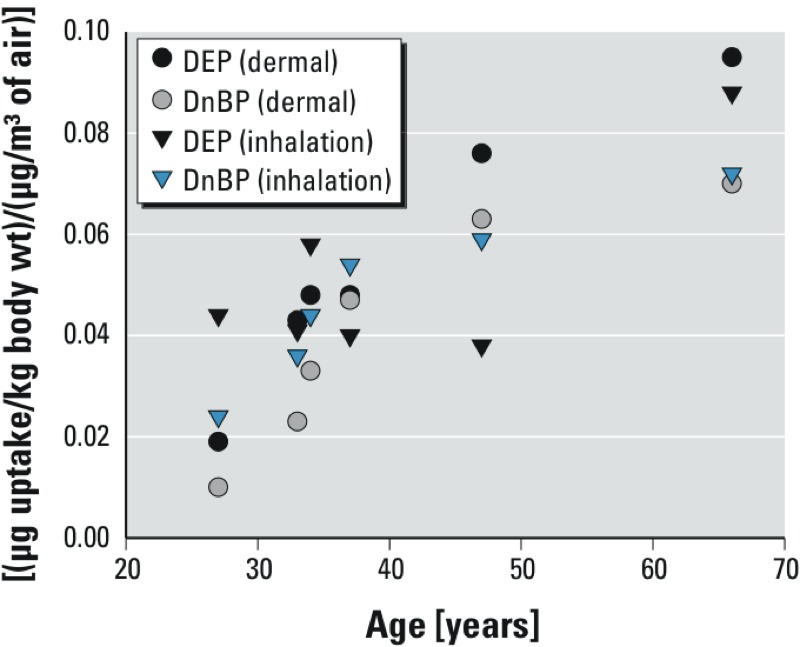
Dermal and inhalation uptakes from chamber air, corrected for uptakes occurring outside the chamber and from hood air, and normalized by chamber air concentrations and body weight plotted against the age of the six participants.

*Differences with age*. In Figure 4, the normalized dermal and inhalation uptakes from air (micrograms of uptake/kilogram body weight per microgram per cubic meter of air) are plotted against the age of the participants. The older the participant, the higher were the dermal uptakes of both DEP and DnBP from air. Based on this limited sample of six, the impact of age is surprisingly strong. The uptake of DEP by the 66-year-old is five times greater than that of the 27-year-old, while the uptake of DnBP is seven times greater. The inhalation pathway also shows increasing uptake with increasing age for DnBP, but the trend is less pronounced for DEP. In the case of the dermal uptake, Spearman’s correlation coefficients (ρ) between uptake and age are 0.9856 (*p* = 0.0003) and 1.0 (*p* = 0.0000) for DEP and DnBP, respectively; in the case of the inhalation uptake, ρ = 0.0286 (*p* = 0.9572) for DEP and ρ = 1.0 (*p* = 0.0000) for DnBP.Although their power is weak, these correlation coefficients support expectations based on visual inspection of Figure 4. They indicate that the trend for increased dermal uptake with increasing age is significant for DEP and DnBP, whereas the trend for increased inhalation uptake is significant for DnBP, but not DEP.

## Discussion

*Comparison of uptake via the dermal pathway with intake via inhalation pathway*. Even when participants wore hoods, the urinary concentrations of MEP, MnBP, and 3OH-MnBP (Figure 1), as well as the net amount of these metabolites excreted over time (Figure 2), increased significantly as a consequence of a 6-hr exposure to elevated air concentrations of DEP and DnBP. Increases were much higher than could be explained by DEP and DnBP in hood air (see Supplemental Material, Table S4). In addition, when participants wore hoods, the total uptakes of DEP and DnBP estimated from urinary metabolite levels—corrected for background and hood air uptakes and normalized by air concentrations (Figure 3) as well as both air concentrations and body weight (see Supplemental Material, Figure S4)—indicate substantial dermal uptake of DEP and DnBP directly from chamber air.

*Volume of air inhaled during 6-hr exposure*. In Figure 3, where the uptake amounts (micrograms) were normalized by the measured air concentrations (micrograms per cubic meter), the resulting units are cubic meters. For inhalation intakes, this value should correspond to the cubic meters of air inhaled during the 6-hr exposure in the chamber. The median values shown for inhalation intakes (3.8 m^3^ for DEP and 3.9 m^3^ for DnBP) are remarkably close to the value predicted for an adult male at rest and breathing at a rate of 0.7 m^3^/hr (U.S. EPA 2011) during a 6-hr period (4.2 m^3^).

*Comparison with predicted absorption from air*. Although these experiments indicate substantial dermal uptake directly from air for both DEP and DnBP, the measured values for the contribution of the dermal pathway directly from air are lower than those predicted in recent studies (Weschler and Nazaroff 2012, 2014; Gong et al. 2014b). The ratio of dermal uptake to inhalation intake predicted by Weschler and Nazaroff (2012, 2014) are based on a steady-state model. However, dynamic modeling (Gong et al. 2014b) indicated that more than 48 hr would be required to reach steady state in the instance of DEP and DnBP; the participants were exposed to elevated concentrations in the chamber for only 6 hr. At the time the participants left the chamber, a large fraction of the DEP and DnBP absorbed by the skin (> 97%) was predicted to be still in the skin. In addition to diffusing into the dermal capillaries, the DEP and DnBP in the skin can desorb to air and the clothing the participants put on after exiting the chamber. Bathing is another sink. Therefore, in the present study we asked participants not to shower for at least 24 hr after leaving the chamber, but this did not preclude hand washing. Desorption from skin to air and clothing, from the time the participants left the chamber until the time the last urine sample was collected, coupled with washing, would meaningfully reduce the dermal uptake of DEP and DnBP. Gong et al. (2014b) did not take clothing and washing into account in their transient model; thus, their model overpredicts the amount of DEP and DnBP that reaches the blood for the period from when participants leave the chamber until their last urine sample.

Dermal absorption occurred both on the exposure days that the participants wore hoods and the days without hoods. The lag between dermal absorption and uptake into the blood is apparent from the fact that, although the urine concentrations of MEP, MnBP, and 3OH-MnBP peaked shortly after the participants left the chamber, the urine concentrations were still two or more times greater than background 40 hr after leaving the chamber. Such a lag between exposure and excretion was not observed when a participant ingested a known amount of labeled DnBP (Koch et al. 2012), nor would it be anticipated for inhalation of DEP or DnBP.

Under typical indoor conditions, occupants tend to be much closer to steady state because they are continuously exposed to DEP and DnBP in home and work environments. Note that the chamber concentrations were roughly two orders of magnitude higher than typical indoor levels so we could distinguish chamber exposures from daily life exposures (e.g., Fromme et al. 2004; Rudel et al. 2003; Wensing et al. 2005; Weschler and Nazaroff 2008). On the other hand, clean clothing may retard dermal uptake from air, and bathing may remove DEP and DnBP temporarily sorbed in the stratum corneum. In other words, although people may be closer to steady-state conditions in daily life, resulting in larger ratios of dermal-to-inhalation uptakes than measured in the present study, other exposure variables may have an opposing influence.

*Differences with age*. The finding that DEP and DnBP dermal uptake was larger for older participants than for younger participants (Figure 4) was unanticipated. The skin’s barrier function is influenced by both its thickness and its lipid content. Although the number of epidermal skin cell layers is similar in older and younger humans, older skin has a thinner epidermis (i.e., it is more compact) and has less lipids than younger skin (Harvell and Maibach 1994; WHO 2006). The net effect of these changes on dermal absorption remains unclear. Roskos et al. (1989) applied various chemicals, radiolabeled with ^14^C, to the inside surface of the forearms of younger (22–40 years) and older (> 65 years) human participants. Absorption was assessed using standard radiotracer methodology on urine samples collected for a week following application. Dermal penetration of four hydrophilic compounds (hydrocortisone, benzoic acid, acetylsalicylic acid, and caffeine) was significantly lower in the older participants than in the younger participants, whereas dermal penetration of two lipophilic compounds (testosterone and estradiol) was not statistically different between the groups. Both DEP and DnBP are lipophilic compounds. In a review of dermal absorption in aged skin, Harvell and Maibach (1994) concluded that opinions regarding differences in percutaneous absorption between young and old skin “are far from a consensus,” and experiments addressing this issue remain non-definitive. This is also the position summarized in a WHO Environmental Health Criteria document on dermal absorption (WHO 2006). We found no studies in the peer-reviewed literature that examined dermal uptake of organic vapors directly from air as a function of age. We recognize that the strong relationship shown in Figure 4 may be due to random error or uncontrolled sources of bias, and we recommend further studies addressing this issue because it has implications for risk assessments in older populations.

*Metabolism of phthalates in skin*. Using viable skin samples and diffusion cells, Beydon et al. (2010) measured dermal permeation and metabolism of DnBP in samples from humans and other animals. They found that carboxylesterases in skin hydrolyzed DnBP to MnBP in all of the species studied and that inhibition of carboxylesterase activity retarded passage of DnBP through the skin. In the case of DEHP, a high-molecular-weight phthalate, Hopf et al. (2014) found that it was metabolized to the monoester MEHP in viable human skin and that MEHP, rather than the parent phthalate, passed into receptor fluid in a diffusion cell. Modeling has indicated that for DEHP, the viable epidermis presents greater resistance to transport than does the stratum corneum; conversely for DEP and DnBP, resistance across the stratum corneum is greater than across the viable epidermis (Weschler and Nazaroff 2012). This is significant because if metabolism occurs in the viable epidermis but not the stratum corneum, it will have a larger impact on the overall rate of dermal uptake for DEHP than for DEP and DnBP. Hopf et al. (2014) assumed that DEHP first permeates the stratum corneum and is then hydrolyzed, arguing that cutaneous esterases are generally not present in the stratum corneum. Beydon et al. (2010) measured carboxylesterase activity in the epidermis and found that it was only 4.2% of that in whole skin. Although this finding does not distinguish between metabolism in the stratum corneum and viable epidermis, it does indicate greater metabolism after DnBP has passed through the epidermis (i.e., stratum corneum and viable epidermis). If, indeed, there is very little metabolic activity in the stratum corneum, then in terms of risk assessment it probably does not matter a great deal whether hydrolysis of DEP and DnBP occurs before or after the phthalate enters the blood. However, such a conclusion requires that the relative dermal toxicity of the monoester is comparable to that of the parent compound.

*Impact of clothing*. In the present study the human participants wore only shorts; the rest of their skin was directly exposed to air. A preliminary evaluation of the impact of clothing on these exposures was conducted concurrently with this study and has been described by Morrison et al. (2015).

*Other phthalates*. For dermal uptake directly from air to contribute significantly to total body burden, an organic compound must possess the right physical–chemical properties—a relatively large value for its skin lipid/air partition coefficient (*K*_sc_g_) and a relatively small molecular weight (Weschler and Nazaroff 2012, 2014; Gong et al. 2014b). Although most phthalate esters have values for *K*_sc_g_ that are favorable for dermal absorption from air, higher-molecular-weight phthalates such as butyl benzylphthalate (BBzP), di(2-ethylhexyl) phthalate (DEHP), and di(isononyl) phthalate (DiNP) tend to have low gas-phase concentrations. This results in kinetic constraints on the flux from air to skin; it is too small for dermal uptake from air to be an important pathway for compounds such as DEHP and DiNP. Although DEHP has been measured at high levels in skin wipes (Gong et al. 2014a; Kim HH et al. 2011), this likely reflects transfer to skin via contact with DEHP contaminated surfaces. In summary, dermal absorption directly from indoor air should be included as a contributory exposure pathway in risk assessments of low-molecular-weight phthalates, but this pathway is anticipated to be of decreasing importance with increasing molecular weight.

## Conclusions

This study has demonstrated that for human participants following a 6-hr dermal-only exposure to elevated gas-phase concentrations of DEP and DnBP, the levels of the metabolites MEP, MnBP, and 3OH-MnBP in urine samples collected over the next 2 days were roughly half those measured in urine samples following a 6-hr dermal plus inhalation exposure. Although earlier assessments of human exposure to phthalate esters have included the dermal pathway (e.g., Guo and Kannan 2011; Koch et al. 2013; Wittassek et al. 2011; Wormuth et al. 2006), it is only recently that dermal absorption directly from air has been part of such assessments (Bekö et al. 2013; Gaspar et al. 2014). The present study provides support for including this pathway in risk assessments. However, the ratios of dermal uptake to inhalation intake for DEP and DnBP assumed in the modeling studies of Bekö et al. (2013) and Gaspar et al. (2014) are higher than those measured in the current study. This apparent disparity may reflect the fact that in our study the participants were exposed to elevated levels of DEP and DnBP for only 6 hr, whereas dynamic modeling indicates that > 40 hr would be required to reach steady-state and maximal uptake via the dermal pathway in comparison to the inhalation pathway.

## Supplemental Material

(633 KB) PDF
