# Supplementary material for: Transdermal Uptake of Diethyl Phthalate and Di(n-butyl) Phthalate Directly from Air: Experimental Verification
Source: Environ Health Perspect. 2015 Apr 7;123(10):928–34. doi: 10.1289/ehp.1409151 (PMC4590762; doi:10.1289/ehp.1409151)
Supplement: (633 KB) PDF [file ehp.1409151.s001.acco.pdf]

Environ Health Perspect
doi: 10.1289/ehp.1409151

Note to Readers: *EHP* strives to ensure that all journal content is accessible to all readers. However, some figures and Supplemental Material published in *EHP* articles may not conform to 508 standards due to the complexity of the information being presented. If you need assistance accessing journal content, please contact ehp508@niehs.nih.gov. Our staff will work with you to assess and meet your accessibility needs within 3 working days.

Supplemental Material

Transdermal Uptake of Diethyl Phthalate and Di(*n*-butyl) Phthalate Directly from Air: Experimental Verification

Charles J. Weschler, Gabriel Bekö, Holger M. Koch, Tunga Salthammer, Tobias Schripp, Jørn Toftum, and Geo Clausen

Table of Contents

Table S1. Chemical and physical properties of DEP and DnBP. All data from Mackay et al. (2006) for 298 K (25°C).

Table S2. Group assignments and physiological parameters for the six male participants.

Table S3. Chamber parameters and experimental conditions.

Table S4. Calculated doses of DEP and DnBP during the six hour chamber exposure without a hood and while wearing a hood (highlighted columns), as well as the background data used to calculate these doses. See *Background and hood air corrections; normalizations* in *Methods* section of main text for additional details.

Figure S1. Co-author G. Bekö wearing breathing hood with latex neck sleeve.

Figure S2. Group 1 participants in the chamber while wearing hoods.

Figure S3. Chamber concentrations of DEP and DnBP during the exposure experiments.

Figure S4. Box-whisker plots displaying the uptakes (µg) of DEP and DnBP, corrected for uptakes occurring outside the chamber and from hood air, normalized by measured chamber air concentrations during each exposure experiment (µg/m^3, Table 1) as well as participants body weight (kg body wt; Table S2), for exposures without a hood (total), exposures with a hood

(dermal) and the differences between these two uptakes (inhalation). Boxes extend from the 25th to the 75th percentile, horizontal bars represent the median, and whiskers indicate the 10th and 90th percentiles. The markers indicate individual results for each of the six participants.

References

Table S1. Chemical and physical properties of DEP and DnBP. All data from Mackay et al. (2006) for 298 K (25°C).

**Abbreviation**	**DEP**	**DnBP**
Chemical name	diethyl phthalate	di(n-butyl) phthalate
Chemical structure	$C_{12}H_{14}O_4$	$C_{16}H_{22}O_4$
CAS-no.	84-66-2	84-74-2
Molar weight (g/mol)	222.24	278.34
Melting point (°C)	-40.5	-35
Boiling point (°C)	295	340
Density (g/ml)	1.12	1.04
Log K_{OW} value	2.47 (median of 13 data)	4.57 (median of 18 data)
Log K_{OA} value	7.55	8.54
Henry constant (Pa m³/mol)	$4.9 \cdot 10^{-2}$ (median of 5 data)	$1.3 \cdot 10^{-1}$ (median of 9 data)
Vapor pressure (Pa)	$8.6 \cdot 10^{-2}$ (median of 9 data)	$3.3 \cdot 10^{-3}$ (median of 25 data)

Table S2. Group assignments and physiological parameters for the six male participants.

Participant	Group	Age (years)	Height (m)	Weight (kg)	BMI[a]	BSA (m^2)[b]
**P1**	1	33	1.83	99	29.6	2.21
**P2**	1	47	1.87	90	25.7	2.16
**P3**	1	66	1.70	63	21.8	1.73
**P4**	2	34	1.80	74	22.8	1.93
**P5**	2	27	1.84	80	23.6	2.03
**P6**	2	37	1.80	77	23.8	1.96

[a]BMI (Body Mass Index) = weight/height^2 (kg/m^2). [b]BSA (Body Surface Area) = 0.20247 · height (m)$^{0.725}$ · weight (kg)$^{0.425}$ (DuBois and DuBois, 1916).

Table S3. Chamber parameters and experimental conditions.

**Parameter**	**Exposure chamber**
Volume, V [m^3]	55
Area, A_s [m^2]	91
Area/volume, A_s/V [m^2/m^3]	1.7
Air flow rate, Q [m^3/h]	39
Material surface, A_m [m^2]	12
Area specific air flow rate, q [$m^3/(m^2$ h)]	3.2
Loading, L [m^2/m^3]	0.22
Air exchange rate, λ [1/h]	0.7

Table S4. Calculated doses of DEP and DnBP during the six hour chamber exposure without a hood and while wearing a hood (highlighted columns), as well as the background data used to calculate these doses. See *Background and hood air corrections; normalizations* in *Methods* section of main text for additional details.

Exposed in chamber without hood

	MEP	DEP dose			MnBP	3-OH MnBP	DnBP dose		
Participant	Total excreted (0-54 h) (µg)	Total (0-54 h) (µg)	Outside chamber (6-54 h) (µg)	Inside chamber (0-6 h) (µg)	Total excreted (0-54 h) (µg)	Total excreted (0-54 h) (µg)	Total (0-54 h) (µg)	Outside chamber (6-54 h) (µg)	Inside chamber (0-6 h) (µg)
P1	1900	2590	160	2435	650	82	1000	180	820
P2	2240	3055	81	2975	1020	120	1560	31	1530
P3	2485	3385	26	3360	910	50	1315	57	1260
P4	1380	1880	62	1820	530	32	770	115	655
P5	935	1275	97	1175	255	32	390	82	310
P6	1170	1590	17	1575	590	62	890	9	880

Exposed in chamber with hood

	MEP	DEP dose				MnBP	3-OH MnBP	DnBP dose			
Participant	Total excreted (0-54 h) (µg)	Total (0-54 h) (µg)	Outside chamber (6-54 h) (µg)	Hood air (0-6 h) (µg)	Inside chamber (0-6 h) (µg)	Total excreted (0-54 h) (µg)	Total excreted (0-54 h) (µg)	Total (0-54 h) (µg)	Outside chamber (6-54 h) (µg)	Hood air (0-6 h) (µg)	Inside chamber (0-6 h) (µg)
P1	1020	1390	145	170	1080	275	38	430	130	24	280
P2	1565	2130	250	170	1710	590	57	885	170	24	695
P3	1245	1695	25	170	1500	440	24	640	70	24	545
P4	905	1230	60	170	1005	310	22	450	85	24	345
P5	550	750	140	170	440	160	14	240	105	24	110
P6	1000	1360	145	170	1045	485	31	705	180	24	505

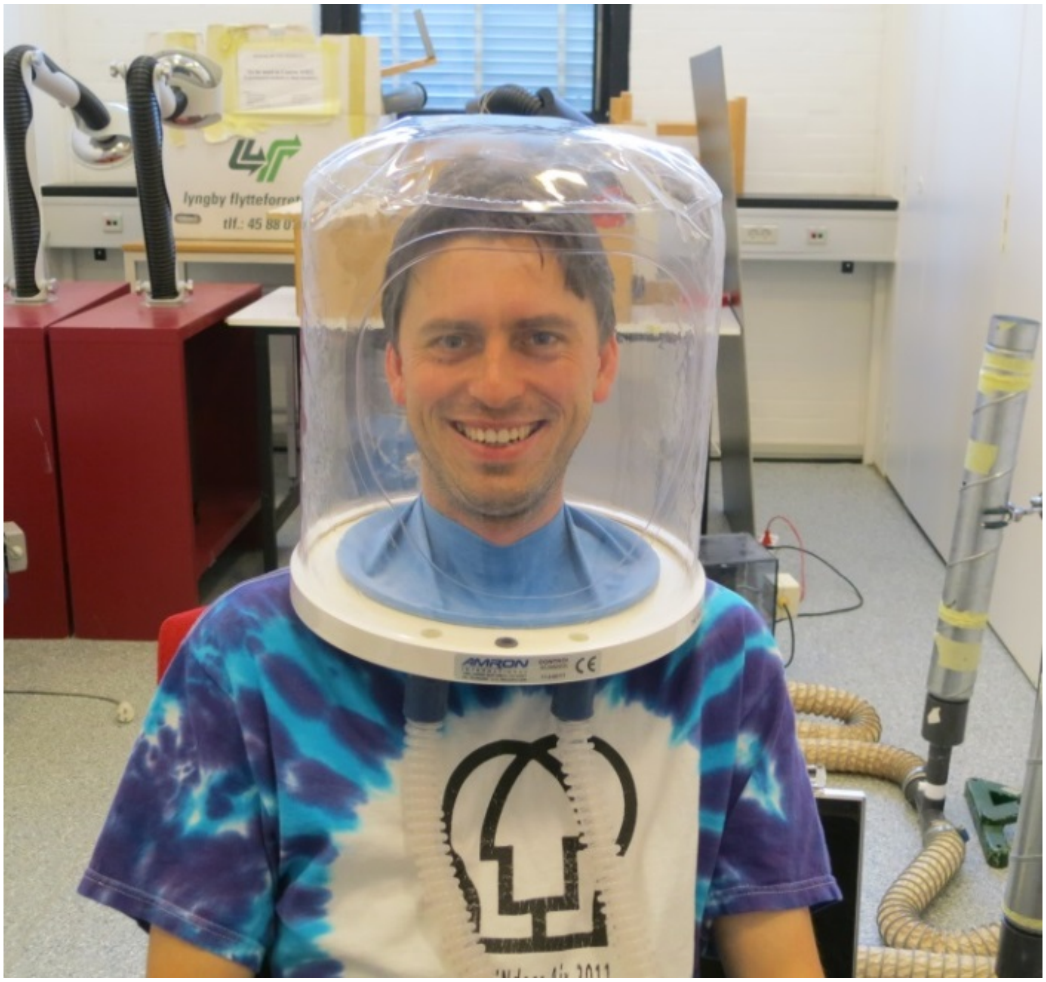

Figure S1. Co-author G. Bekö wearing breathing hood with latex neck sleeve.

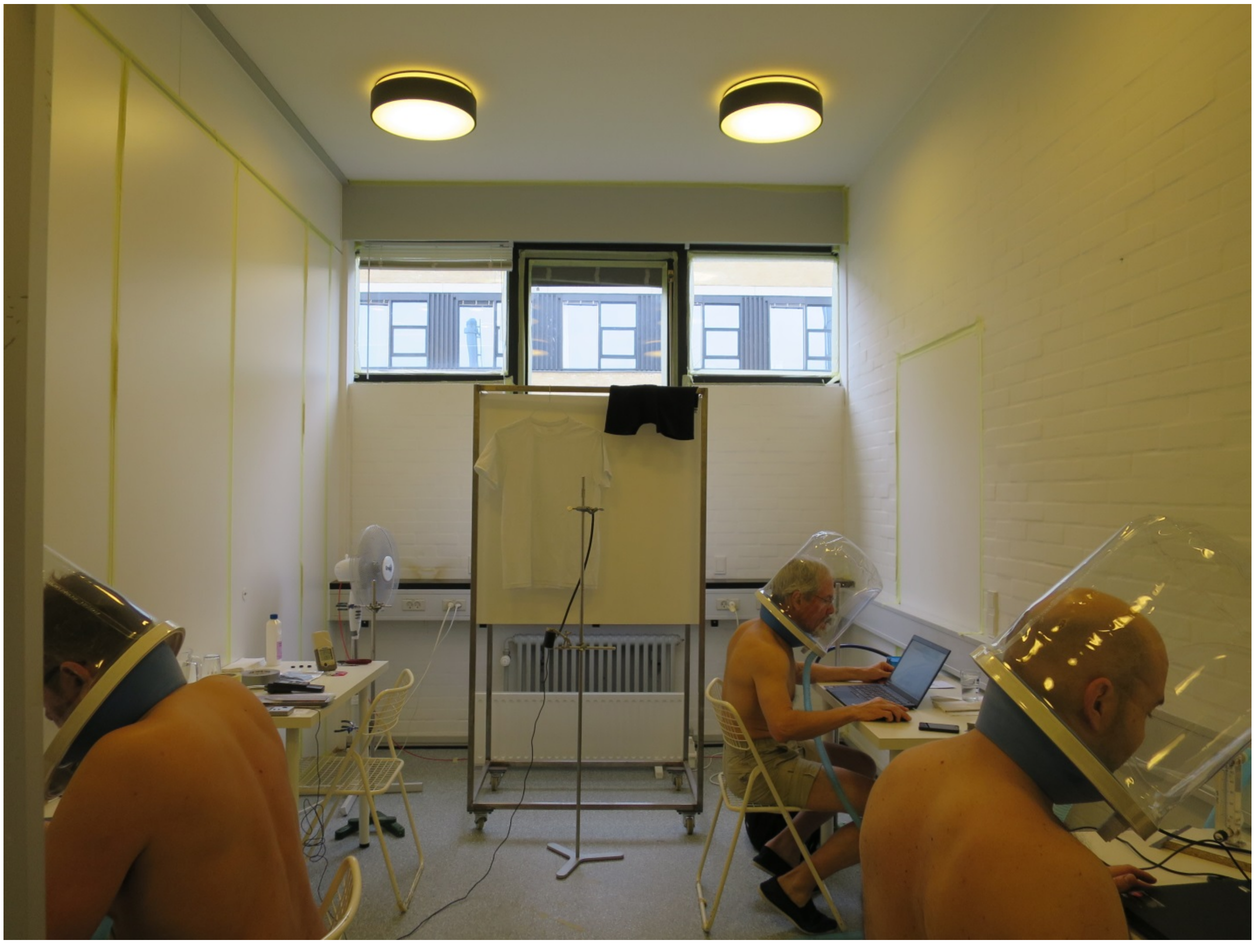

Figure S2. Group 1 participants in the chamber while wearing hoods.

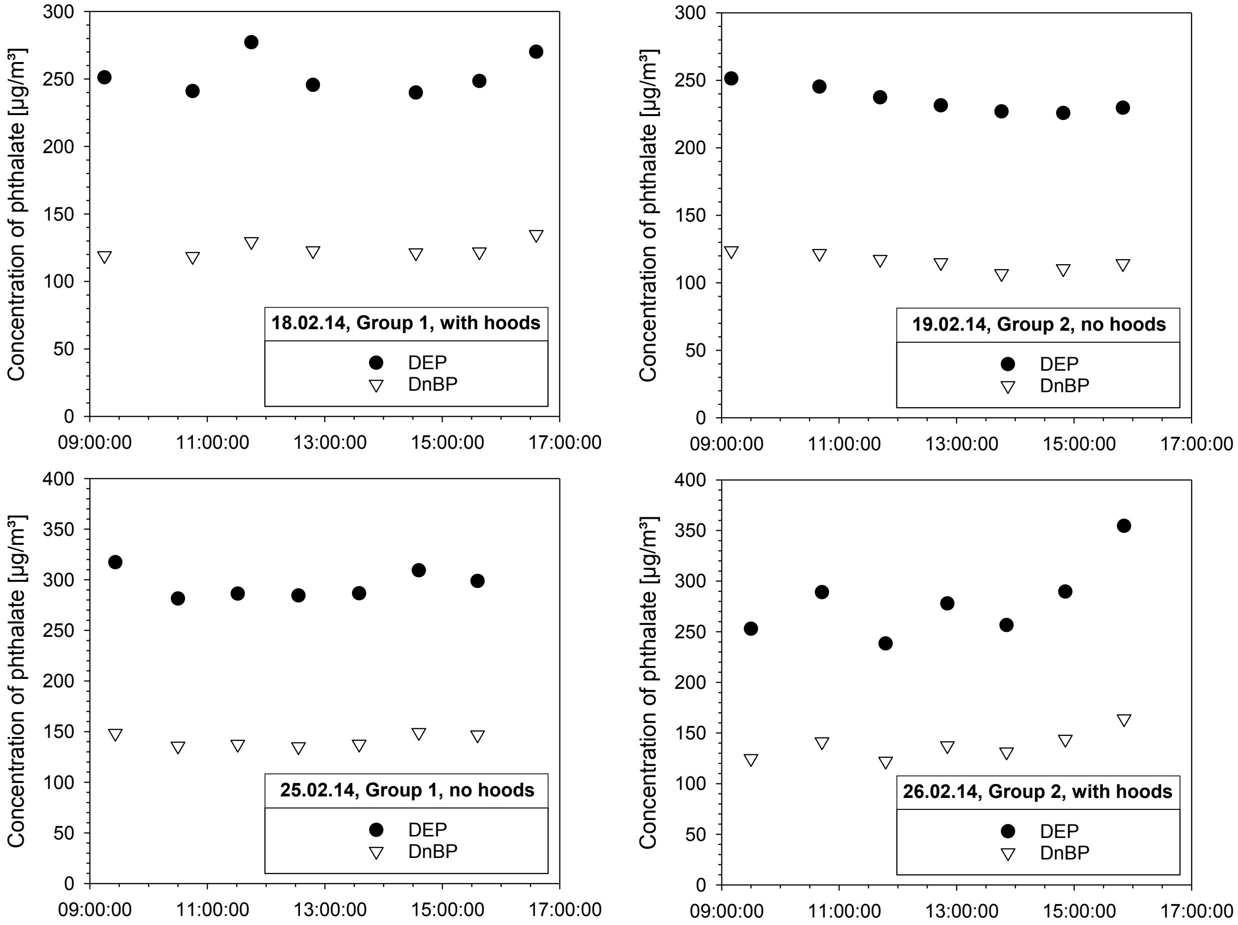

Figure S3. Chamber concentrations of DEP and DnBP during the exposure experiments.

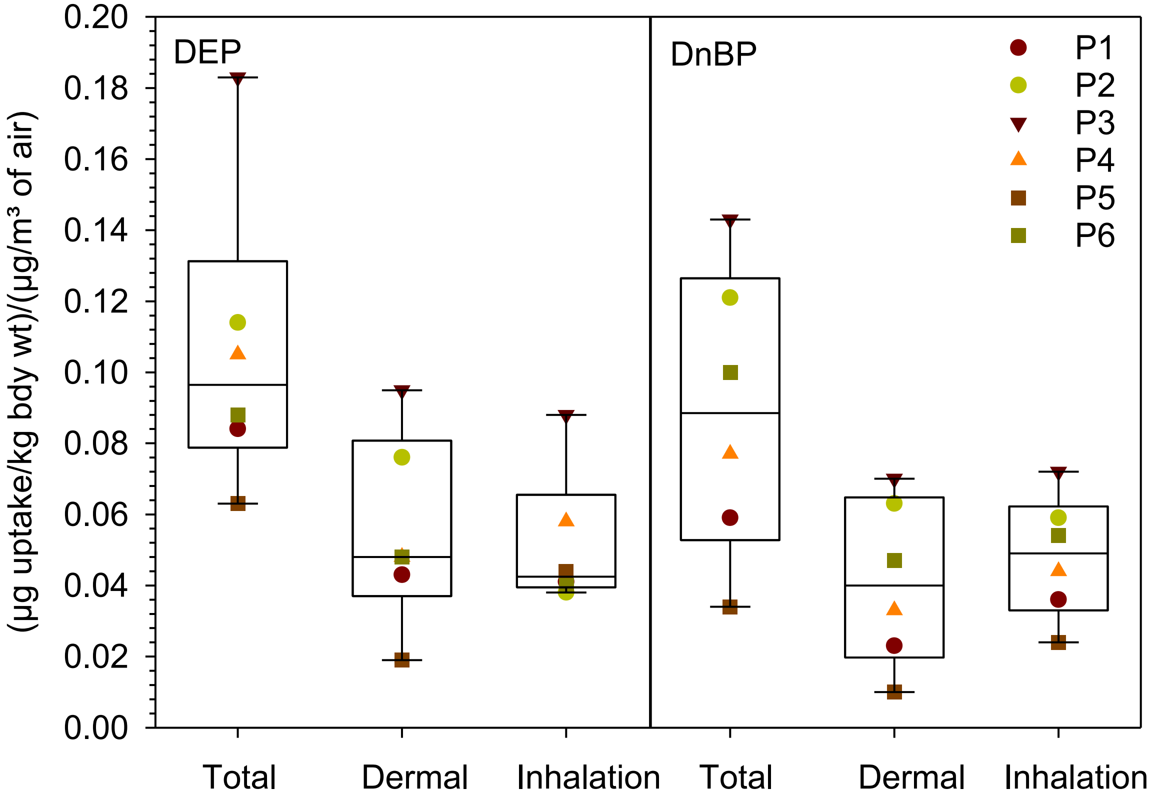

Figure S4. Box-whisker plots displaying the uptakes (µg) of DEP and DnBP, corrected for uptakes occurring outside the chamber and from hood air, normalized by measured chamber air concentrations during each exposure experiment (µg/m^3, Table 1) as well as participants body weight (kg body wt; Table S2), for exposures without a hood (total), exposures with a hood (dermal) and the differences between these two uptakes (inhalation). Boxes extend from the 25th to the 75th percentile, horizontal bars represent the median, and whiskers indicate the 10th and 90th percentiles. The markers indicate individual results for each of the six participants.

References

DuBois D, DuBois EF. 1916. A formula to estimate the approximate surface area if height and weight be known. Arch Intern Medicine 17:863-71.

Mackay D, Shiu WY, Ma K-C, Lee SC. 2006. Handbook of Physical-Chemical Properties and Environmental Fate for Organic Chemicals, Volume III – Oxygen Containing Compounds. Boca Raton, FL: CRC Press, Taylor & Francis Group.
